# A Review of Foreign Language Learners’ Emotions

**DOI:** 10.3389/fpsyg.2021.827104

**Published:** 2022-01-10

**Authors:** Qiangfu Yu

**Affiliations:** Faculty of Humanities and Foreign Languages, Xi’an University of Technology, Xi’an, China

**Keywords:** foreign language anxiety, foreign language enjoyment, emotional intelligence, foreign language learners, positive psychology

## Abstract

In the past half century, the research on emotions of foreign language learners (FLL) has shifted the focus from an exclusive analysis of negative emotions to a more holistic study of both negative emotions and positive emotions, and currently to mediators of multiple emotions. Of the FLL’s negative emotions, foreign language anxiety (FLA) attracts the most attention. Researchers have widely discussed the relationship between FLA and foreign language achievement, the influencing factors of FLA, the dynamicity of FLA as well as regulation and intervention strategies of FLA. Foreign language enjoyment (FLE) is the most heavily studied research subject on FLL’s positive emotions. Researchers generally focus on the influencing factors of FLE, the dynamicity of FLE and the relationship between FLE and FLA. With the implementation of holistic education, emotional intelligence (EI), a mediator of multiple emotions of FLL, has been becoming a newly hot topic in the field of FLL’s emotions. By reviewing the previous studies, this paper proposes that the future research on FLL emotion needs to expand the research perspectives, enrich the research topics, and innovate the research methods.

## Introduction

As an activity to promote the development of learners’ cognitive and thinking abilities, foreign language learning can stimulate learners to experience a variety of emotions, and emotions have an inseparable relationship with cognitive ability, which is an important factor affecting foreign language learning. However, the roles of emotions in foreign language learning had long been relatively ignored. In the 1970s, researchers began to study FLL’s emotions (Chastain, 1975; Horwitz et al., 1986; Liu and Jackson, 2008; Marcos-Llinás and Garau, 2009; Dewaele and MacIntyre, 2014; Dewaele, 2019; Dewaele et al., 2019; MacIntyre et al., 2019). In the past half century, anxiety and enjoyment, the two most common FLL’s emotions, have attracted the most attention (Dewaele and MacIntyre, 2014, 2016; Li et al., 2018). The recent development and expansion of positive psychology in second language acquisition has broken the previous situation of over-focusing on negative emotions like anxiety in research on FLL’s emotions, and FLL’s positive emotions have begun to draw attention from scholars (Dewaele and MacIntyre, 2014, 2016; Dewaele and Alfawzan, 2018; Elahi Shirvan et al., 2020; Elahi Shirvan and Taherian, 2020). The educational concept of paying equal attention to negative and positive emotional experiences has provided a new perspective for the study on FLL’s emotions (Dewaele and Dewaele, 2017; Boudreau et al., 2018; Elahi Shirvan and Talebzadeh, 2018a,c; Elahi Shirvan and Taherian, 2021). Furthermore, attention has been paid to individual differences and dynamic complexity in FLL’s emotions (Ushioda, 2015; Larsen-Freeman, 2016; Dewaele and Dewaele, 2017; Boudreau et al., 2018; Elahi Shirvan and Talebzadeh, 2018a,b, 2020; De Ruiter et al., 2019; Lowie and Verspoor, 2019; Talebzadeh et al., 2020; Elahi Shirvan and Taherian, 2021). Meanwhile, since research on one single emotion is not enough to account for the differences of FLL on foreign language learning, EI, which can regulate negative emotions, generate and make full use of positive emotions, promote critical thinking (Ciarrochi and Mayer, 2007), and maximize the effect of foreign language learning (Pishghadam, 2009), began to attract attention from researchers (Abdolrezapour and Tavakoli, 2012; Ponikwia, 2015; Shakarami and Khajehei, 2015; Li et al., 2020; Mavrou and Dewaele, 2020; Resnik and Dewaele, 2020; Irini, 2021; Nasim and Parisa, 2021). This paper reviews the research on FLL’s emotions, summarizes its development and major findings in the past five decades, and presents the prospect of future research on FLL’s emotions.

## Foreign Language Learners’ Negative Emotions

Foreign language learning is a complex cognitive activity with rich emotional experiences, during which FLL experience various negative emotions such as anxiety, fear, tension, shame, burnout, anger, and boredom (Horwitz et al., 1986; Teimouri, 2018; Kruk, 2019; Li, 2020a). These negative emotions will affect the optimal learning potential of FLL, and in many cases weaken the language learning competence of FLL. Of the negative emotions of FLL, FLA has attracted the most attention (Dewaele and MacIntyre, 2014). In this review paper, FLA refers to either foreign language anxiety or second language anxiety.

### Definitions of Foreign Language Anxiety

As a most significant learners’ emotional factor, anxiety has become a focus in research on language acquisition since the 1970s. The term of anxiety, originating from psychology, refers to “an unpleasant state of mind that is characterized by individual perceived feelings like nervous, fear, and worry, and is activated by the autonomic nervousness system” (Spielberger, 1972). The development of Foreign Language Classroom Anxiety Scale (FLCAS) by Horwitz et al. (1986) aroused growing attention to FLA. FLA was conceptualized as a unique form of anxiety specific to foreign language learning context (Horwitz et al., 1986; MacIntyre, 1995). Horwitz et al. (1986) proposed that FLA is “a distinct complex of self-perceptions, beliefs, feelings, and behaviors related to classroom language learning arising from the uniqueness of the language learning process.” MacIntyre and Gardner (1991) defined FLA as the feeling of tension and apprehension specifically associated with a context requiring the use of a foreign language that the individual is not proficient with. Aida (1994) held the opinion that FLA is unique to the language learning process and there are fear and nervousness in self-consciousness, emotions, beliefs, and behaviors related to classroom language learning. Recently, with the application of dynamic approach to FLA, FLA was regarded as an emotion of multi-faceted nature and in a fluctuating state over time, even from second to second and from minute to minute (Saghafi and Elahi Shirvan, 2020) and the fluctuation of FLA was reported to be affected by contextual factors (Gkonou, 2017; Saghafi et al., 2017).

### Foreign Language Anxiety and Foreign Language Achievement

The development of FLCAS by Horwitz et al. (1986) is of great significance to research on FLA. A large number of research has found a consistent inverse relationship between FLA and foreign language achievement (MacIntyre and Gardner, 1991; Horwitz, 2001; Liu and Jackson, 2008). With the deeper understanding of FLA, some researchers came to realize that FLL experience anxiety not only in classroom, but also in the processes of acquiring all the foreign language skills, such as listening, speaking, reading, writing, translating, and interpreting. FLA at the level of specific language skills is different from foreign language classroom anxiety in that FLA can more directly reflect the relationship between anxiety and various foreign language skills (Cheng et al., 1999). Therefore, scholars have successively developed FLA scales for various foreign language skills, such as Foreign Language Oral Anxiety Scale (Woodrow, 2006), Foreign Language Reading Anxiety Scale (Saito et al., 1999), Foreign Language Listening Anxiety Scale (Elkhafaifi, 2005), Foreign Language Writing Anxiety Scale (Cheng, 2004), etc.

Although an overwhelming majority of quantitative studies have shown that learners’ FLA can negatively predict foreign language achievement (Aida, 1994; Onwuegbuzie et al., 2000; Salehi, 2014), some qualitative studies have found that moderate FLA can facilitate foreign language learning (Scovel, 1978; Marcos-Llinás and Garau, 2009). Alpert and Haber (1960) classified anxiety into facilitating anxiety and debilitating anxiety, the former influencing students in a positive and motivating way and sometimes regarded as enthusiasm in performing a given task, while the later bringing students some unpleasant feelings and words which could negatively affect students’ academic performance in foreign language learning. And there are some other studies revealing that there is no correlation between FLA and foreign language achievement (Chastain, 1975; Zhang, 2013). Thus, FLA does not always inhibit learners’ foreign language learning efficacy, and its impact on foreign language learning is complex. Moreover, FLA may be affected by other individual or social factors. Therefore, multiple variables should be incorporated to have a better comprehension of FLA.

### Influencing Factors of Foreign Language Anxiety

As an individual subjective emotional experience, FLA is closely related to a large number of individual and social factors. Previous studies have found that the major factors affecting learners’ FLA include learners’ age, gender, personality, discipline background, mother tongue proficiency, overseas learning experience, self-efficacy, learning motivations, willingness to communicate in foreign language, learning strategies, teachers’ characteristics, teaching methods, and classroom environment (Oxford, 1999; Teimouri et al., 2019). The relationship between some variables and FLA is relatively clear, and the research conclusion are relatively consistent. For example, extroverted learners are more willing to communicate in foreign language classroom and their FLA level is relatively lower (Krashen, 1982); leaners with negative learning attitudes are more likely to experience relatively higher level of FLA (Trylong, 1987; MacIntyre and Gardner, 1994); learners with richer overseas learning experience tend to experience lower FLA level (Coleman, 1997); learners proficient in employing foreign language learning strategies are more likely to experience lower FLA level (Liu and Thondhlana, 2015); learners more willing to communicate or having stronger motivations experience lower level of FLA (Hu, 2016).

However, the relationship between some variables and FLA seems to be more complex, and great differences have been found in some relevant studies. For example, some scholars have found that male learners’ FLA is higher than that of female learners (Lu and Liu, 2015), some believe that female learners’ FLA is higher than that of male learners (Elkhafaifi, 2005), and some have pointed out that gender is not significantly correlated to FLA (Shi and Fan, 2013). A few studies have found that the subject background of FLL is significantly correlated with FLA level: compared with FLL majoring in humanities, learners specializing in natural sciences have a higher level of FLA (Wang T., 2014), while some researchers hold the opinion that there is no significant correlation between learners’ subject background and their FLA (Tsai and Lee, 2018).

The reasons for these differences may be attributed to the differences in regional social culture, gender concepts, and research methodologies adopted by researchers. However, this, to a certain extent, hinders the development of research on FLA intervention, making it difficult to effectively implement in the practice of foreign language teaching some suggestions advocated by researchers, such as group teaching and layered teaching. Therefore, the exploration of the influencing factors of FLA and its mechanisms needs to be further deepened.

### Dynamicity of Foreign Language Anxiety

Language learning is an emotionally and psychologically dynamic process influenced by a multitude of changing variables producing moment-by-moment fluctuations in learners (Gregersen et al., 2014), making FLA dynamically changing over time (Dewaele and Dewaele, 2017; Dewaele and Alfawzan, 2018; Dewaele et al., 2017; Elahi Shirvan and Taherian, 2021). Dynamicity of FLA is supported by several empirical studies conducted in recent years. Saghafi et al. (2017) took an ecological analysis of English as a foreign language (EFL) learners’ writing anxiety within the framework of nested ecosystem model. Through the methods of semi-structured stimulated recall interviews, questionnaire, teacher observation, student journal, and task-motometer instrumented over ten classroom sessions, four upper-intermediate learners’ writing anxiety was examined from the perspectives of micro-, meso-, exo-, and macro-systems. Findings of the study showed that EFL learners’ writing anxiety fluctuates within the interactive relation of the individual and environmental variables.

Similarly, based on nested ecosystems model and complex dynamic system theory, Kasbi and Elahi Shirvan (2017) conducted a study on dynamicity of EFL learners’ speaking anxiety. Findings of their research indicated that EFL learners’ speaking anxiety fluctuates in classroom and that learners’ level of speaking anxiety is affected differently by the events within the dynamics of classroom ecology.

Additionally, Saghafi and Elahi Shirvan (2020), applying an idiodynamic method, investigated topic-based fluctuations in FLA with the subjects of two low-anxiety learners and two high-anxiety learners selected from 20 female intermediate EFL learners on the basis of FLCAS assessments. Outcomes of the research revealed that FLA fluctuates from topic to topic and four major factors as linguistic block, topic familiarity, topic interest, and topic-related emotional loading affect the dynamicity of FLA.

### Intervention of Foreign Language Anxiety

Shi and Xu (2013), after reviewing the achievements made in research on FLA in recent 40 years, pointed out that empirical intervention research on learners’ FLA is very scarce. However, due to the development of interdisciplinary research and modern technology media, such research has been greatly enriched nowadays. Specifically, on the one hand, some scholars intervene learners’ FLA from the perspective of cognitive science and psychology. For example, inspired by the psychological outpatient technology for the treatment of general social anxiety, Wang M. (2014) carried out cognitive reconstruction on 32 first-year non-English majors by way of Rational Emotive Behavior Therapy (REBT), verifying the applicability of REBT technology to lower learners’ foreign language oral anxiety. Meng and Chen (2014) intervened a college student’s FLA through Cognitive Behavioral Therapy and found that the FLA level of the subjects decreased significantly after the intervention. Jin et al. (2021) took a positive psychology approach to investigate whether reminiscing about language achievements could effectively decrease the learners’ foreign language classroom anxiety, by taking 88 Chinese students of English as the subjects. It was revealed that both dimensional and overall levels of anxiety diminished significantly in the experimental group after a 30-day intervention.

On the other hand, with the development of new media technology, especially after the outbreak of COVID-19, the teaching environment has gradually moved from offline classrooms to online classroom, the teaching mode is becoming increasingly diversified, and learners’ learning environment is becoming increasingly autonomous. Some scholars attempted to use network multimedia as the media to intervene learners’ FLA (Chen and Lee, 2011). For example, Zhang (2013) compared learners’ FLA under different center modes in the network multimedia teaching environment and found that the “double center” teaching mode with equal emphasis on “teachers” and “students” has the best effect on alleviating FLA. Wang and Zhang (2021) analyzed the psychological anxiety of students taking a college English online course and proposes some effective methods to decrease the student’s psychological anxiety under the network environment, such as advocating the mode of group learning and peer cooperation, strengthening the timeliness and diversity of tests, increasing the richness of extracurricular activities, and improve teachers’ online teaching quality. Lv et al. (2016), comparing the anxiety of FLL under different communication modes, found that synchronous computing mediated communication has a certain slowing effect on FLA; however, there are some differences in the degree of anxiety of FLL with different foreign language proficiency before and after the intervention. Therefore, it is suggested that while giving full play to the advantages of network multimedia, attention should be paid to the combination of educational technologies and learners’ individual differences.

Reviewing the research on FLA, we can find that the research methods have been greatly enriched: from quantitative analysis to case analysis and then to interactive analysis; the research perspectives have been fully expanded: from traditional classroom to computer-aided situation, and then to network multimedia platform; the research results have been continuously deepened: from understanding anxiety to coping with anxiety, and then to intervening anxiety. However, there are still many gaps in the study of FLA. For example, researchers do not have a comprehensive knowledge and comprehension of FLA, focusing too much on the inhibitory effect of FLA on foreign language achievement and paying less attention to the positive impact of FLA; focusing too much on FLA at the level of one specific foreign language skill and failing to take into account the cross correlation between FLA and different foreign language skills; the mechanism of multiple factors affecting FLA being still unclear. These gaps need to be filled by a large number of theoretical and empirical studies in the future.

## Foreign Language Learners’ Positive Emotions

With the establishment and development of positive psychology in the 21st century, as well as the introduction of positive psychology to the field of second language acquisition by MacIntyre and Gregersen (2012), research on FLL’s emotions has gradually broken the previous situation dominated by negative emotions and begun to pay more attention to the positive emotions that can enable learners to make achievements, enhance self-efficacy and achieve self-realization (Jiang and Li, 2017), shifting the research focus from an exclusive analysis of negative emotions to a more holistic study of both negative emotions and positive emotions. Of the FLL’s positive emotions of enjoyment/joy, gratitude, hope, pride, inspiration, well-being, empathy, mindfulness (Snyder and Lopez, 2009; MacIntyre et al., 2019), FLE has drawn the greatest amount of attention from researchers (Dewaele and MacIntyre, 2014, 2016; Elahi Shirvan et al., 2021).

### Definitions of Foreign Language Enjoyment

Foreign language anxiety refers to the positive emotions that learners feel after overcoming learning difficulties, completing academic tasks, and realizing psychological needs in the process of foreign language learning (Dewaele and MacIntyre, 2014; Dewaele et al., 2016). Boudreau et al. (2018) distinguished enjoyment from pleasure, a more basic positive emotional experience, in that “if pleasure can occur simply by performing an activity or completing an action, enjoyment takes on additional dimensions such as an intellectual focus, heightened attention, and optimal challenge.” As one of the most prevalent and significant positive emotions experienced by FLL across different contexts (Pavelescu and Petric, 2018; Piniel and Albert, 2018), FLE can be positively mediated by interactions with peers who are friendly and communicative, teachers who are supportive and encouraging, and classroom activities which are interesting and adequately challenging (Dewaele and MacIntyre, 2014; Pavelescu and Petric, 2018). Moreover, based on the Foreign Language Enjoyment Scale developed by Dewaele and MacIntyre (2014), a large number of studies conducted in foreign language acquisition in diverse cultures reveal that FLE can help FLL have a better understanding and acquisition of a foreign language (Dewaele and Alfawzan, 2018; Dewaele and Dewaele, 2018; Saito et al., 2018; Li, 2020b).

### Influencing Factors of Foreign Language Enjoyment

As an individual subjective emotion, FLE is also affected by a series of individual and social variables, including learners’ gender, age, multi-lingual background, foreign language learning attitude, teacher’s characteristics, and classroom environment (Dewaele et al., 2017, 2019). It is found that the older FLL are, the higher their FLE level is, and that female FLL have a higher level of FLE than male FLL. For example, Dewaele and MacIntyre (2014) surveyed 1746 FLL from all over the world and found that learners who have mastered multiple languages, have a higher level of foreign language, feel more skilled than their peers, have a higher level of education and are older in age have a higher FLE level. Dewaele and Dewaele (2017) studied the levels of FLE and FLA of 189 British middle school students. It was found that both FLE level and FLA level of female learners are higher than male learners, and that female learners’ emotional integration in FLL is also higher.

Another study (Dewaele et al., 2017) pointed out that learners’ internal variables (such as age, gender, foreign language proficiency, learning attitude, etc.) have a higher correlation with their FLE and FLA, while external variables (especially those related to teachers, such as teachers’ code switching between foreign language and first language, teachers’ classroom behavior, etc.) have a lower correlation with FLE and FLA. Further research also found that classroom variables related to teachers have a higher correlation with learners’ FLE, but a lower correlation with FLA.

More notably, Ross and Rivers (2018) discussed the emotional experience of eight college English learners in various social interactions outside the classroom and found that their emotional experience outside the classroom is particularly strong compared with the formal language learning in classroom and that learners are more likely to experience FLE if they can successfully use the language outside the classroom.

Elahi Shirvan and Taherian (2020) investigated the actualization of FLE within the microsystem of the classroom of a listening and speaking course by way of a social hermeneutics approach from an ecological perspective. The findings highlight that teachers is a contributing factor to the emergence of FLE. Besides, other ecological factors, such as learners’ characteristics, rules and regulations of the university, also contribute to the emergence of FLE in FLL.

These findings shake the argument that teachers can significantly lower learners’ FLA by adjusting classroom teaching behavior, which has been repeatedly emphasized by researchers for a long time. They also inspire teachers not to exaggerate learners’ negative emotions, but to improve learners’ experience of positive emotions.

### Dynamicity of Foreign Language Enjoyment

Like FLA, FLE was also demonstrated by some studies to be a dynamic concept. Dewaele and Dewaele (2017) took a pseudo-longitudinal investigation of how FLE of FLL evolved over time. It was reported that the causes of FLE change over time and that the mean value of FLE increases over time, indicating the dynamicity of FLE. Moreover, Elahi Shirvan and Talebzadeh (2018a) employed an idiodynamic method to investigate the moment-to-moment fluctuations of FLE in seven female university students and found that FLE changes dynamically among individuals from topic to topic as learners have reported different degrees of FLE throughout the conversation process in the classroom. Their findings contribute to the investigation of underlying factors of the dynamicity of FLE. Similarly, Li et al. (2018) assessed fluctuations in FLE and FLA in Anglo-Canadian learners of French as a second language and found that both fluctuate severely second by second. The complex and dynamic nature of FLE was supported again by Elahi Shirvan and Talebzadeh (2018b) when they explored to what extent FLE is transparent to teachers and peers.

The dynamic turn in the field of educational psychology has inspired more researchers to explore the dynamics of FLE by utilizing different techniques. De Ruiter et al. (2019) used the technique of Kohonen’s Self-Organizing Maps to investigate the level of teacher’s emotional support in alignment with its relationship with FLE and FLA. It was illustrated that teacher support, FLE and FLA fluctuate throughout the interaction process between the teacher and the student, who are deemed to be dynamic systems. Elahi Shirvan and Talebzadeh (2020) used the technique of Retrodictive Qualitative Modeling to analyze the signature dynamics of FLE and FLA. They found that both FLE and FLA fluctuates periodically under the influences of different attractor states. Besides, Elahi Shirvan et al. (2020) employed a time-based sampling scheme of ecological momentary assessment to explore the dynamism of FLE among EFL learners across different time scales. Their findings indicated that FLE fluctuates from moment to moment, from week to week and also from month to month. Furthermore, to investigate the dynamic features of FLE among EFL learners, Elahi Shirvan et al. (2021) used a longitudinal confirmatory factor analysis-curve of factors model to analyze the extent of changes in FLE at different points of time. They observed that FLL with the initially lower level of FLE experiences more changes over time while FLL with the initially higher level of FLE experiences less change.

### Relationships Between Foreign Language Enjoyment and Foreign Language Anxiety

The correlation between FLE, a representative of positive emotions, and FLA, a representative of negative emotions, is also a topic of academic discussion. On the one hand, some scholars pointed out that learners’ FLE is significantly negatively correlated with FLA, which means that the higher the learners’ FLE is, the lower their FLA level is (Dewaele and MacIntyre, 2014). Therefore, enhancing learners’ FLE experience seems to be an effective means to alleviate learners’ FLA. However, some scholars believe that although FLE and FLA seem to be negatively correlated in some cases, they are not in the relationship of “binary opposition” and “one falling and another rising,” but coexisting in FLL’s learning process (Dewaele and MacIntyre, 2016; Boudreau et al., 2018). For example, Boudreau et al. (2018) explored the dynamic relationship between FLE and FLA in classroom by taking 10 English native speakers in French learning as the research object. It was shown that the interaction between learners’ positive emotions and negative emotions is complex: they can be significantly negatively correlated (e.g., higher FLE is accompanied by lower FLA), or significantly positively correlated (e.g., higher FLE is accompanied by higher FLA), or not significantly correlated. It can be seen that multiple foreign language emotions may act on learners’ foreign language learning process, and emotions themselves are affected mutually by a series of complex factors. Therefore, the investigation of a single emotion is not enough to fully explain learners’ foreign language learning efficacy, and understanding learners’ foreign language learning process with the coexistence of multiple emotions has become a new research interest.

## Emotional Intelligence—Mediator for Foreign Language Learners’ Multiple Emotions

In recent years, inspired by relevant theories in the field of educational psychology, scholars in the field of second language acquisition began to pay more attention to FLL emotions, such as shame, boredom, burnout, pride, courage, etc. (Teimouri, 2018; Pawlak et al., 2020a; Shao et al., 2020), and the relevant scales are becoming growingly mature. At the same time, some scholars recognized that there is no absolute distinguishment of good and bad between positive emotions and negative emotions, and that both can have positive or negative effects (Shao et al., 2020). With the deepening understanding of the coexistence of multiple emotions in learners’ foreign language learning process, researchers began to explore learners’ multiple emotion processing mechanisms. Jiang and Li (2017) believe that all individuals will experience various emotional changes, but that their ability to deal with emotions, that is, EI, varies from individual to individual.

Emotional intelligence refers to the ability of individuals to perceive, evaluate and express emotions, to approach and understand emotions, and to regulate emotions to promote emotional and intellectual development (Mayer and Salovey, 1997). It was found that EI can significantly alleviate and regulate negative emotions like FLA, which has an indirect positive impact on learners’ foreign language achievements (Pishghadam, 2009; Yu et al., 2015). Moreover, with the help of structural equation model, mediation model, regression analysis, and other statistical means, researchers further found that emotions play an intermediary role between EI and academic achievements (Li, 2020a). For example, by examining the relationships among emotional intelligence, FLA, and English achievements in 510 Chinese non-English-major students, Yu et al. (2015) find that the students’ EI is negatively correlated with their FLA but positively linked with their English achievements and that FLA has partial mediating effect on EI in predicting students’ English achievements and self-rating English proficiency. Besides FLA, there are some other emotions having mediating effect on EI. Li (2020a) investigated the relationships of EI and the three emotions of anxiety, enjoyment and burnout with English achievements with 1307 Chinese Year-2 senior high school students as the subjects, and results show that enjoyment, anxiety, and burnout co-mediate the significant relationship between EI and English achievement, the mediating effect of burnout being the strongest and the mediating effect of enjoyment being the weakest.

To sum up, it can be found that foreign language learning is not only affected by learners’ single emotion, but affected by a combination of multiple emotions and multiple factors. If the integrity of learners’ emotional experiences were ignored and focus were only laid on the impact of a single emotion on learners’ academic achievements, we would not fully understand learners’ learning emotional experiences, nor can we give learners enough “whole person” care. In addition, individual emotions are diverse, changing and complex. In the foreign language teaching environment where individual differences are common, it is by no means easy to implement unified intervention on FLL’s emotions, but it is practical and feasible to cultivate learners’ ability to understand, perceive and regulate their own emotions. However, the current academic research on the mediators of FLL’s multiple emotions has just begun, and the empirical research on “EI regulating emotions” is particularly lacking, which are expected to become the focus of future research.

## Suggestions for Future Research

Research on FLL’s emotions has shifted the focus from an exclusive analysis of negative emotions to a more holistic study of both negative emotions and positive emotions in the past half century. We suggest the future research on FLL’s emotions should seek breakthroughs from the following respects: the expansion of research perspectives, the enrichment of research topics and the innovation of research methods.

### Expanding Research Perspectives

Dewaele (2005) pointed out that the introduction of interdisciplinary theories and methods is conducive to the further development of research on emotions in second language acquisition. Therefore, research on FLL’s emotions should be further combined with the relevant theoretical achievements of other disciplines, especially positive psychology, neurolinguistics, ecology, and computing science. Besides, current research on FLL’s emotions mainly focuses on college students and postgraduates, and research on primary and secondary school students as well as ethnic minority students is in a relatively blank state. Consequently, future research on FLL’s emotions should pay more attention to the groups mentioned above. For example, there are many Confucius Institutes worldwide, but research on emotions of learners of Chinese as a second language has not been reported, which is worth exploring. Furthermore, most of the intervention strategies proposed in the research on FLL’s emotions are explored from the perspective of teachers, but there are few studies on how FLL intervenes with emotions. Investigation of what intervention strategies FLL adopts and what differences are found in the use of these strategies between FLL with higher level of emotion(s) and FLL with lower level of emotion(s) from the perspective of students instead of teachers is to be explored. Finally, since emotions like FLA and FLE are complicated and dynamic, Dörnyei and Ryan (2015) proposed the necessity to adopt dynamic research approaches to reveal the changing nature of emotions of FLL during the acquisition process. And for this purpose, longitudinal perspectives should be considered in future research on FLL’s emotions (Elahi Shirvan and Taherian, 2021).

### Enriching Research Topics

Research topics on FLL’s emotions should be enriched in future study. Firstly, most previous studies only focus on the overall foreign language emotion of FLL or the emotional experience at a specific language skill level, which often leads to conflicting or even contradictory research conclusion. However, various foreign language skills are not independent of each other, and the learners’ emotional experiences promoted by different foreign language skills interact on each other, synthesizing the overall emotional experience of FLL. Therefore, when exploring the emotional experience of FLL at the level of specific language skill, researchers may also consider the correlation between different language skills and conduct cross, comprehensive and multi-factor research on different language skills. Secondly, considering that the foreign language negative emotion (such as anxiety) scales for different language skills are relatively sophisticated while the positive emotion scales at different language skills are still almost blank, future research can further develop and refine the foreign language positive emotion scales. And more attention should be paid to the cross influence of positive and negative emotions and the index weight comparison of different dimensions of emotions, so as to provide effective suggestions for intervention on FLL’s emotions in foreign language teaching. Thirdly, the relationship between external environmental factors and FLL’s emotions should be stressed. With the increasing popularity of online teaching, especially under the background of diversified teaching forms in the “post-epidemic era,” attention should be paid not only to the online teaching and classroom teaching environment, but also to the extracurricular environments rarely mentioned in the previous research. Based on the previous research on the relationship between teachers’ teaching style, teacher-student relationship and FLL’s emotions, further concern should be extended to the effects of teaching materials, teachers, teaching software, and hardware environments during online teaching on FLL’s emotions, as well as the relationship between parents, activity design and other factors in extracurricular teaching environment and FLL’s emotions. Fourthly, because FLL’s emotions are generated in interaction (Pawlak et al., 2020b), learners’ interaction with peers, teachers and multimodal learning environment in the learning process should also be emphasized, so as to better understand the generation of FLL’s emotions under different conditions and their relationship with other factors and to have a more comprehensive grasp of FLL’s emotions as a whole. For example, as a participant in the teaching-learning process, the effects of teachers’ thoughts, emotions and behaviors on students’ psychology and learning efficacy cannot be ignored (Xu, 2020). Therefore, the relationship between teacher-student interaction and FLL emotions may become a focus of future research. Finally, the research on FLL’s emotions in the past 50 years mainly discusses the complex relationship between FLL’s emotions and foreign language achievements, and few studies are involved in how to intervene FLL’s emotions. Future research can try to explore the construction of intervention model of FLL’s emotions from multiple dimensions through such statistical methods as cluster analysis, structural equation modeling, mediating effect test, and multiple logistic regression.

### Innovating Research Methods

We believe that future research on FLL’s emotions should innovate its research methods, so as to keep abreast of the expansion of research perspectives and the enrichment of research topics. FLL’s emotions are a complex multi-dimensional system, which arises from the interaction between learners and the ecological environment, and has the characteristics of dynamics and complexity, and researchers have already begun to explore the dynamics of FLL’s emotions, but the current research is far from enough. To demystify the dynamics of both positive and negative emotions of FLL, more studies should be conducted with reference to some newly developed research methods from the ecological perspective of complex system theory, such as idiodynamic method (Elahi Shirvan and Talebzadeh, 2018a; Saghafi and Elahi Shirvan, 2020), clustering technique of Self-Organizing Maps (De Ruiter et al., 2019), retrodictive qualitative modeling (Elahi Shirvan and Talebzadeh, 2020), ecological momentary assessment (Elahi Shirvan et al., 2020). Besides, with regard to the development of quantitative research questionnaires, combined with the characteristics of different learning groups, localized FLL’s emotions questionnaires highlighting the characteristics of foreign language learnin g should be developed with reference to the sophisticated foreign language anxiety scale, such as FLCAS by Horwitz et al. (1986).

## Conclusion

This paper reviews the development of research on FLL’s emotions. Generally speaking, in the past 50 years, research on FLL’s emotions has experienced a focus shift from an exclusive analysis of negative emotions to a more holistic study of both negative emotions and positive emotions, and currently to mediators of multiple emotions. The review paper analyzed the research status of positive emotions and negative emotions with the representatives of FLA and FLE, as well as EI. Based on the analysis, this paper looks forward to the future research prospects, and believes that further attention should be paid to expanding the theoretical perspectives, enriching the research topics and innovating the research methods. FLL’s emotions are a complex multi-dimensional system, which needs more in-depth exploration and research by scholars in future.

## Author Contributions

The author confirms being the sole contributor of this work and has approved it for publication.

## Conflict of Interest

The author declares that the research was conducted in the absence of any commercial or financial relationships that could be construed as a potential conflict of interest.

## Publisher’s Note

All claims expressed in this article are solely those of the authors and do not necessarily represent those of their affiliated organizations, or those of the publisher, the editors and the reviewers. Any product that may be evaluated in this article, or claim that may be made by its manufacturer, is not guaranteed or endorsed by the publisher.
